# Machine Learning Identification of Neutrophil Extracellular Trap-Related Genes as Potential Biomarkers and Therapeutic Targets for Bronchopulmonary Dysplasia

**DOI:** 10.3390/ijms26073230

**Published:** 2025-03-31

**Authors:** Xuandong Zhang, Bingqian Yan, Zhou Jiang, Yujia Luo

**Affiliations:** Department of NICU, Sir Run Run Shaw Hospital, Zhejiang University School of Medicine, Hangzhou 310016, China

**Keywords:** bronchopulmonary dysplasia, machine learning, immune infiltration, biomarkers, neutrophil extracellular traps

## Abstract

Neutrophil extracellular traps (NETs) play a key role in the development of bronchopulmonary dysplasia (BPD), yet their molecular mechanisms in contributing to BPD remain unexplored. Using the GSE32472 dataset, which includes 100 blood samples from postnatal day 28, we conducted comprehensive bioinformatics analyses to identify differentially expressed genes (DEGs) and construct gene modules. We identified 86 DEGs, which were enriched in immune and inflammatory pathways, including NET formation. Weighted gene co-expression network analysis (WGCNA) revealed a key gene module associated with BPD. By intersecting 69 NET-related genes (NRGs), 149 module genes, and 86 DEGs, we identified 12 differentially expressed NET-related genes (DENRGs). Immune infiltration analysis revealed an increase in neutrophils, dendritic cells, and macrophages in BPD patients. Machine learning models (LASSO, SVM-RFE, and RF) identified 5 upregulated biomarkers—MMP9, Siglec-5, DYSF, MGAM, and S100A12—showing potential as diagnostic biomarkers for BPD. Validation using nomogram, ROC curves, and qRT-PCR confirmed the diagnostic accuracy of these biomarkers. Clinical data analysis showed that Siglec-5 was most strongly correlated with BPD severity, while DYSF correlated with the grade of retinopathy of prematurity (ROP) and its laser treatment. Clustering analysis revealed two distinct BPD subtypes with different immune microenvironment profiles. Drug–gene interaction analysis identified potential inhibitors targeting MGAM and MMP9. In conclusion, the study identifies five NET-related biomarkers as reliable diagnostic tools for BPD, with their upregulation and association with disease severity and complications, such as ROP, highlighting their clinical relevance and potential for advancing BPD diagnostics and treatment.

## 1. Introduction

Bronchopulmonary dysplasia (BPD) represents a significant chronic pulmonary disorder predominantly affecting preterm infants, particularly those with extremely low birth weight (ELBW) [1]. Although prenatal steroid use, postnatal surfactant supplementation, and enhanced ventilation techniques have advanced, the incidence of BPD continues to rise with increased survival of extremely premature infants [2,3]. BPD is characterized by arrested alveolar development, simplified alveolar structure, and persistent inflammation, leading to long-term respiratory morbidity and increased mortality [4].

The pathogenesis of BPD is complex and multifactorial, with inflammation playing a central role. Recent studies have highlighted the importance of innate immunity, especially neutrophils, in the development of BPD as the primary host defense mechanism [5]. Neutrophil extracellular traps (NETs), first described by Brinkmann in 2004, represent a novel mechanism by which neutrophils combat pathogens by releasing extracellular fibrous networks comprised of DNA, histones, and granular proteins [6]. Although NETs play a vital role in eliminating pathogens, their improper regulation is associated with numerous inflammatory disorders. Growing evidence suggests that NETs may contribute to BPD pathogenesis. Baraldi et al. have shown that NET components, including neutrophil elastase and myeloperoxidase, are elevated in the bronchoalveolar lavage fluid of premature infants who develop BPD [7]. Furthermore, experimental models have demonstrated that NETs can promote lung injury through multiple mechanisms, including direct tissue damage, activation of inflammatory pathways, and interference with normal lung development [8]. Moreover, Li et al. have revealed that NETs can affect lung development through the WNT/β-catenin signaling pathway, a crucial regulator of alveolar formation [9]. Despite these advances, the molecular mechanisms underlying NET-mediated lung injury in BPD remain incompletely understood. Moreover, there is a lack of reliable biomarkers for early BPD prediction and monitoring.

Advancements in high-throughput sequencing and bioinformatics now enable comprehensive analysis of gene expression profiles to identify disease-specific molecular signatures. This study utilized integrated bioinformatics analysis using both clinical samples and public datasets to identify NET-related genes and molecular signatures in BPD. This study aimed to identify and validate the clinical predictive ability of key molecular biomarkers for bronchopulmonary dysplasia development in premature infants through the application of multiple machine learning technologies, WGCNA methods, and immune cell infiltration analysis while highlighting potential therapeutic targets through drug–gene interaction analysis. Our findings offer fresh understanding into the molecular mechanisms of BPD, potentially aiding in the creation of novel diagnostic and therapeutic strategies.

## 2. Results

### 2.1. Determination and Enrichment Analysis of DEGs

**Figure 1:**
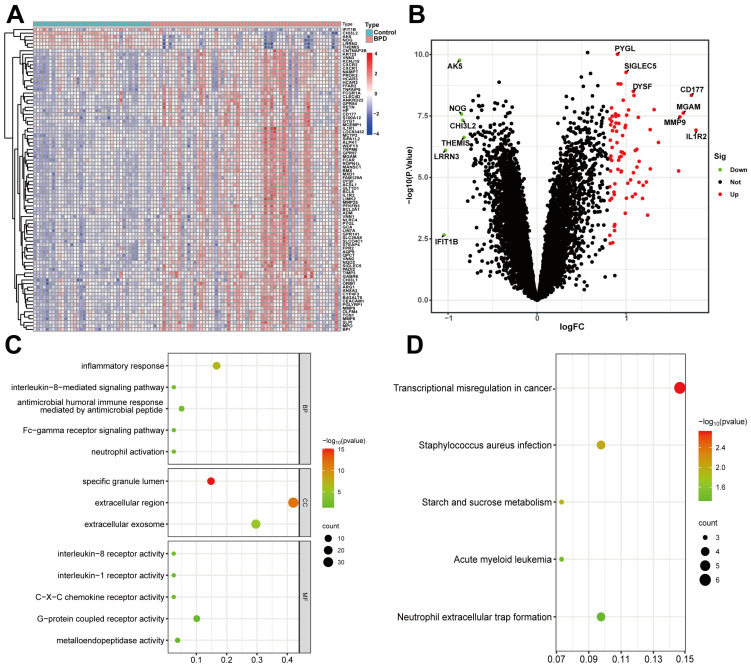
Genes differentially expressed and functional enrichment in BPD neonates and controls. (**A**) Heatmaps and (**B**) volcano plots. (**C**) GO analyses were performed to forecast the prospective functions of DEGs containing CC, BP, and MF. (**D**) KEGG pathways concerning DEGs were assessed.

Among the training samples, 62 patients with BPD and 38 controls carried out DEG analysis. The BPD group exhibited 86 DEGs relative to the control group, with 80 genes upregulated and 6 downregulated. Heatmaps and volcano plots effectively presented the significant findings (Figure 1A,B). DEGs were predominantly enriched in GO and KEGG pathways associated with immune and inflammatory response pathways. The top biological process, cellular component, and molecular function were identified, highlighting functional enrichment in inflammatory responses, specific granule lumen, and interleukin-8 receptor activity in BPD (Figure 1C). Meanwhile, DEGs are primarily enriched in the KEGG pathways of neutrophil extracellular trap formation, transcriptional misregulation in cancer, and staphylococcus aureus infection (Figure 1D).

### 2.2. Gene Module Identification and Co-Expression Network Formation

**Figure 2:**
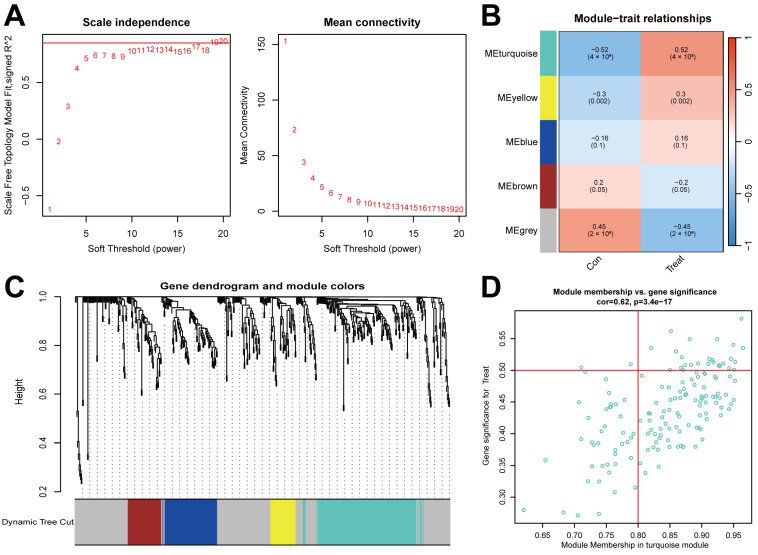
An analysis of WGCNA and identification of possible significant genes. (**A**,**B**) WGCNA’s soft threshold power and mean connectivity. (**C**) Clustering gene dendrogram and modules of WGCNA. (**D**) The interaction genes between DEGs and ME turquoise module.

To identify important gene modules related to BPD, WGCNA analysis was utilized to construct gene modules and co-expression networks for both BPD and control groups. Following the standard WGCNA methodology, we tested various soft threshold values ranging from 1 to 30 using the pickSoftThreshold function. After analysis, a soft threshold of 19 was chosen as it achieved optimal network topology characteristics with a scale-free topology fitting index R^2^ of 0.85, indicating good compliance with scale-free properties (Figure 2A). According to guidelines from WGCNA developers, an ideal scale-free R^2^ value should not be less than 0.80, and our selection represents a balance between maintaining moderate network connectivity while achieving a high R^2^ value, thereby reducing noise connections while preserving sufficient gene interaction information. Using the dynamic cutting algorithm, five co-expression modules (turquoise, blue, brown, yellow, and green) were identified and visualized in the TOM heatmap (Figure 2B–D). The turquoise module showed the strongest correlation with BPD status (r = 0.52, *p* < 0.001) and contained 149 co-expressed genes with distinctive expression patterns between BPD and control samples. Given its robust disease association, we focused subsequent analyses on the turquoise module, particularly examining its relationship with neutrophil extracellular trap formation identified in our initial DEG pathway analysis.

### 2.3. Identifying DENRGs in BPD

**Figure 3:**
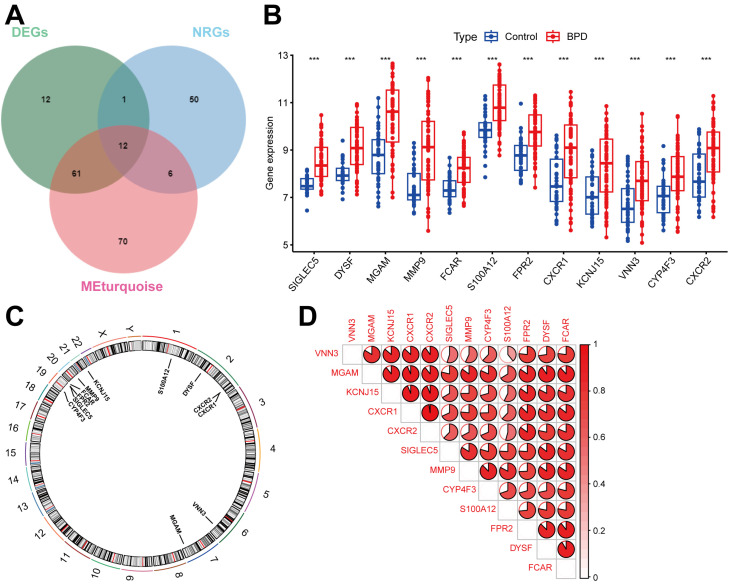
Landscape of 12 DENRGs in BPD. (**A**) Identification of 12 DENRGs. (**B**) Histogram of the expression of 12 DENRGs between BPD patients and controls. (**C**) Chromosomal positions of the 12 DENRGs. (**D**) Spearman’s correlation analysis of 12 DENRGs. Notes: *** *p* < 0.001.

The intersection approach combining NRG-associated genes, turquoise module genes, and DEGs represents a multidimensional strategy to identify the most biologically relevant genes in BPD pathogenesis. This strategy prioritizes genes that are not only differentially expressed but also function within coordinated networks and participate in neutrophil-related processes, helping to pinpoint the most biologically relevant genes in BPD pathogenesis. We identified 12 DENRGs by intersecting 69 NRG-associated genes, 149 genes from the turquoise module, and 86 DEGs, as illustrated in a Venn diagram (Figure 3A). The histogram illustrates significant expression differences of the 12 DENRGs between BPD and control samples. All 12 genes (Siglec-5, DYSF, MGAM, MMP9, FCAR, S100A12, FPR2, CXCR1, KCNJ15, VNN3, CYP4F3, and CXCR2) show statistically significant upregulation in BPD samples compared to controls (Figure 3B, *p* < 0.001). Notably, genes like MGAM, MMP9, and S100A12 display the most pronounced upregulation, with MGAM showing the highest median expression in BPD samples. These consistent expression patterns across all identified neutrophil-related genes strongly suggest their collective involvement in the inflammatory processes characteristic of BPD pathogenesis. In Figure 3C, a circular diagram depicts the chromosomal positions of the 12 DENRGs. Ultimately, a correlation heatmap and Spearman’s analysis revealed that the correlation between CXCR1 and CXCR2 was the strongest and positive (cor = 0.97, *p* < 0.001), with no negative correlations observed (Figure 3D).

### 2.4. Immune Cell Infiltration in BPD

**Figure 4:**
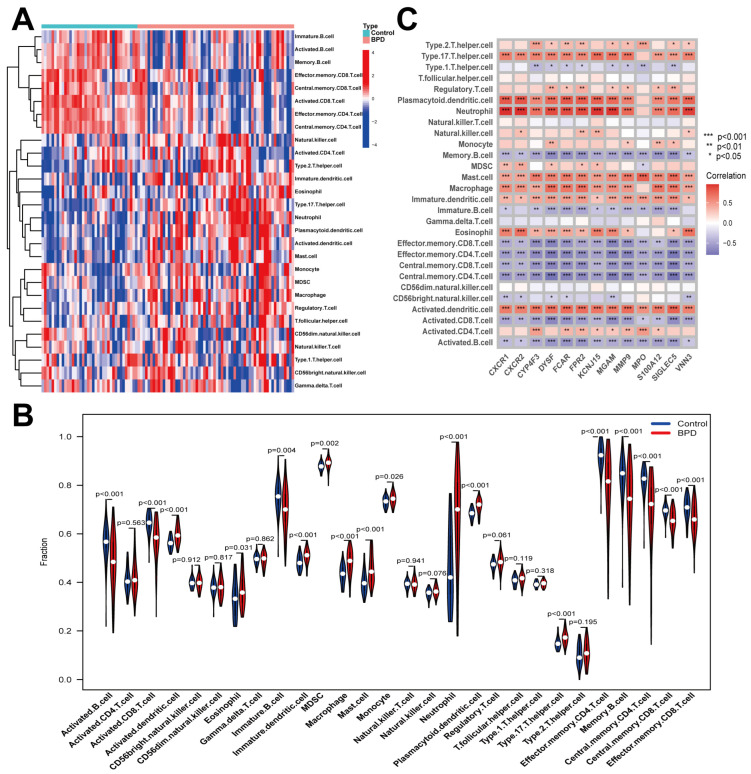
Evaluation of immune cell infiltration. (**A**) By ssGSEA score, the heatmap showed enriched immune cells in every sample. (**B**) A violin graph illustrated the distinct fractions of immune cells in the BPD neonates and controls. (**C**) Correlation between determined genes and immune cells. Notes: * *p* < 0.05, ** *p* < 0.01, *** *p* < 0.001.

Significant differences in the proportions of 28 immune cell types between BPD neonates and controls were uncovered by the ssGSEA method (Figure 4A). Notably, the algorithm discovered vital reductions in the proportion of B cells, CD4 T cells, and CD8 T cells (*p* < 0.001), whereas the proportion of neutrophils, dendritic cells, MDSC, monocytes, and macrophages was increased (*p* < 0.001) in BPD patients in comparison to controls (Figure 4B). In addition, correlation analysis exhibited that the 12 DENRGs were related to neutrophils, dendritic cells, and macrophages (Figure 4C). The findings indicated that DENRGs were crucial in modulating immune cell infiltration and molecular processes in BPD.

### 2.5. Screen Key Biomarkers Machine Learning Models

**Figure 5:**
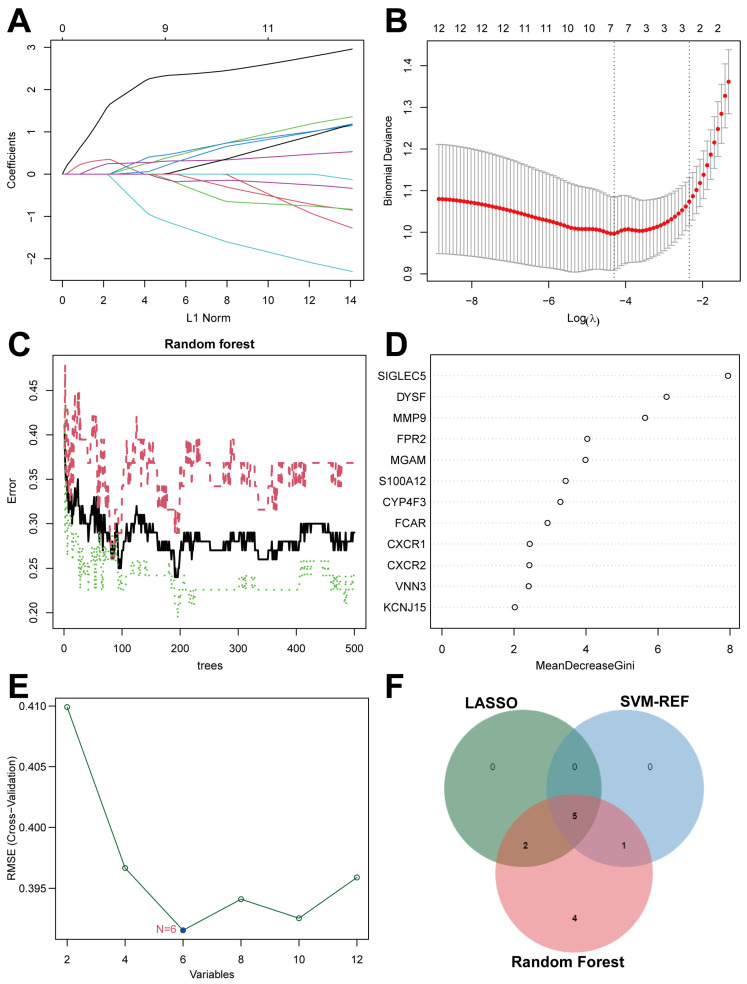
Detection of key biomarkers in BPD infants. (**A**,**B**) Algorithm LASSO identified 7 biomarkers. (**C**,**D**) Algorithm RandomForest algorithm determined 12 biomarkers. (**E**) Algorithm SVM-RFE selected 6 biomarkers. (**F**) Determination of key biomarkers by interacted genes.

LASSO, SVM-RFE, and RF algorithms were applied to evaluate 12 DENRGs for identifying credible diagnostic biomarkers linked to BPD. Initially, we applied the least squares method to integrate the expression patterns of 12 DENRGs into LASSO regression. The study identified 7 potential DENRGs and determined the optimal lambda value (Figure 5A,B). The RF algorithm effectively screened 12 vital genes (Figure 5C,D). Figure 5E demonstrates that the SVM-RFE analysis obtained 6 DENRGs as reliable molecular biomarkers for BPD. In BPD, 5 DENRGs were identified: matrix metallopeptidase 9 (MMP9), sialic acid binding Ig-like lectin 5 (Siglec-5), dysferlin (DYSF), maltase-glucoamylase (MGAM), and S100 calcium-binding protein A12 (S100A12). All biomarkers were found to be upregulated in BPD patients (Figure 5F).

### 2.6. Validation of the Diagnostic Capabilities of Five Biomarkers

**Figure 6:**
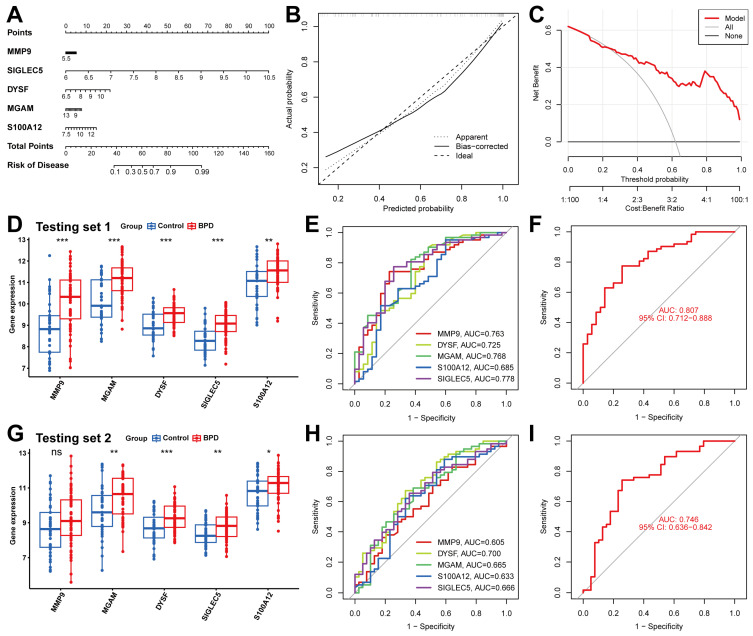
The performance of 5 biomarkers in the training set. (**A**) Construction of a nomogram for predicting the occurrence of BPD based on biomarkers. (**B**) Calibration curve. The y-axis is the actual rate of BPD diagnosis; the x-axis is the predicted risk of BPD. The diagonal dotted line represents a perfect prediction by an ideal model. The solid line represents the bias-corrected performance of the nomogram, where a closer fit to the diagonal dotted line represents a better prediction. (**C**) Decision curve analysis. The red represents the net benefit of the nomogram in the prediction of BPD occurrence. The grey line represents the assumption that all people have BPD. The black line represents the assumption that all people do not have BPD. (**D**) Expression of MMP9, MGAM, DYSF, Siglec-5, and S100A12 in testing set 1. (**E**,**F**) ROC curves of the 5 biomarkers in the BPD test set 1 alone or in combination for diagnosis. (**G**) Expression of MMP9, MGAM, DYSF, Siglec-5, and S100A12 in testing set 2. (**H**,**I**) ROC curves of the 5 biomarkers in the BPD test set 2 alone or in combination for diagnosis. Notes: ns > 0.05, * *p* < 0.05, ** *p* < 0.01, *** *p* < 0.001.

A nomogram was created to assess the risk linked to factors influencing NET formation in 62 BPD patients, facilitating the evaluation of the machine learning algorithm’s predictive accuracy. The higher the expression level of Siglec-5, the higher the diagnostic score of BPD, indicating that the expression level of Siglec-5 was crucial in the diagnosis of BPD (Figure 6A). A moderate disparity between the predicted and actual cluster risk for BPD was shown by the calibration curve (Figure 6B). The DCA results corroborated the nomogram’s high accuracy (Figure 6C), pointing to its value as a resource for directing treatment strategies in BPD. To assess their diagnostic effectiveness, we produced ROC curves for the biomarkers in two testing sets. Differential analysis of peripheral blood data from BPD patients in validation set 1 (5 days post-birth) and validation set 2 (14 days post-birth) revealed a significant increase in 5 markers in the BPD group compared to the control group, with the exception of MMP9 in validation set 2.6D, 6G). The ROC curve analysis indicated an AUC value of approximately 0.75 for the 5 markers in validation set 1, with an overall AUC of 0.807 (Figure 6E,F). The AUC for the 5 markers in validation set 2 was approximately 0.65, with an overall AUC of 0.75 (Figure 6H,I). This showed that the 5 markers do have good diagnostic ability.

### 2.7. Determination of NRG Molecular Clusters in BPD

**Figure 7:**
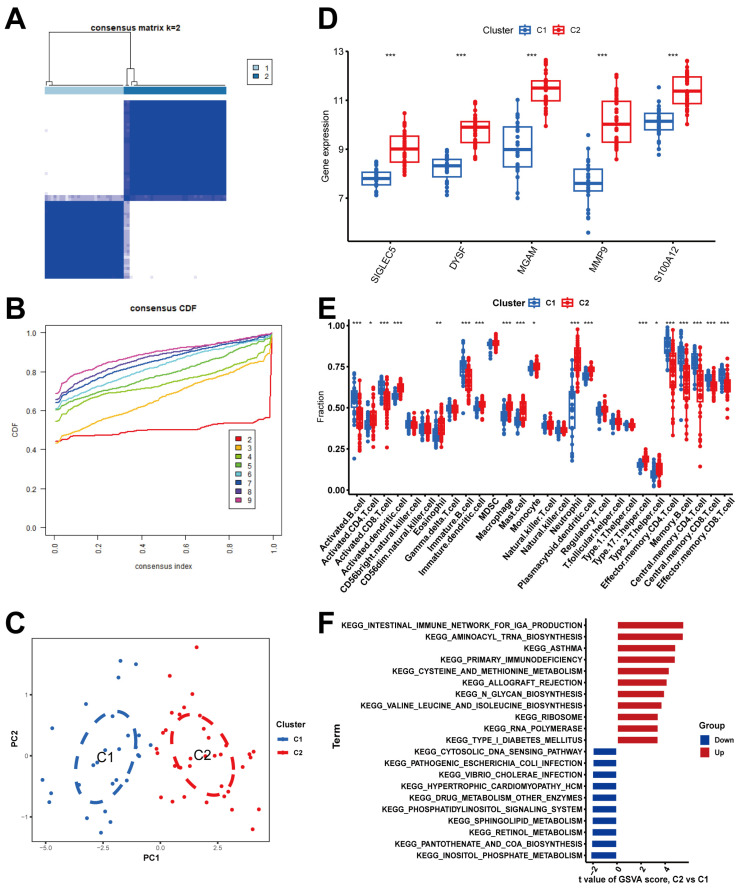
Molecular subtypes characterized based on 12 DENRGs. (**A**) Heatmap of 2 clusters (k = 2) derived from DENRGs. (**B**) Cumulative distribution graph. (**C**) PCA analysis of the 2 clusters: blue indicates cluster 1; red indicates cluster 2. (**D**) Box plots of gene expression levels between two clusters. (**E**) Different expression levels of immune cells between cluster 1 and cluster 2. (**F**) GSVA analysis between cluster 1 and cluster 2. Notes: * *p* < 0.05, ** *p* < 0.01, *** *p* < 0.001.

The consensus clustering analysis revealed robust stratification of BPD patients into two distinct molecular subtypes (Figure 7A). As shown in Figure 7B, the CDF curve demonstrates clear improvement when moving from k = 2 (red line) to higher cluster numbers, with minimal additional benefit beyond k = 3, confirming the stability of our two-cluster model. This optimal clustering is visualized in the consensus matrix showing strong intra-cluster consensus and clear separation between clusters. The PCA plot further validates this classification, with samples from clusters C1 and C2 forming distinct groups in dimensional space (Figure 7C). The histogram indicated that five biomarkers exhibited high expression levels in cluster C2 (Figure 7D). Subsequently, we examined the distinction in immune microenvironment characteristics between two clusters and found that C2 cluster shared the same immune microenvironment as the overall BPD population and was the typical disease subtype (Figure 7E). Functional enrichment analysis indicated that cluster C2 showed upregulation in the pathways related to asthma, primary immunodeficiency, and intestinal immune network for IgA production (Figure 7F).

### 2.8. Drug–Gene Interactions for BPD Patients

**Figure 8:**
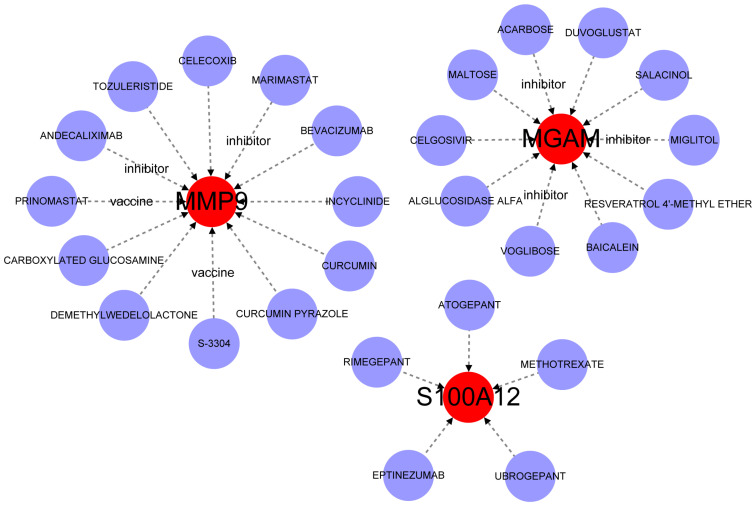
Drug–gene interaction analysis of the 3 biomarkers of BPD. Drug–gene interaction diagram. The red circle indicates the key biomarkers; the purple circle indicates the drug.

Only 3 biomarkers have been predicted with 27 drugs. These validated inhibitors for MGAM included ACARBOSE, MIGLITOL, and VOGLIBOSE. Meanwhile, MMP9 also had two validated inhibitors, like MARIMASTAT and ANDECALIXIMAB, as well as two vaccines called PRINOMASTAT and S-3304. Furthermore, Cytoscape software (v3.10.3) facilitated the visualization of drug-gene interactions (Figure 8).

### 2.9. Relationship Between Biomarkers and Patients’ Clinical Characteristics

**Figure 9:**
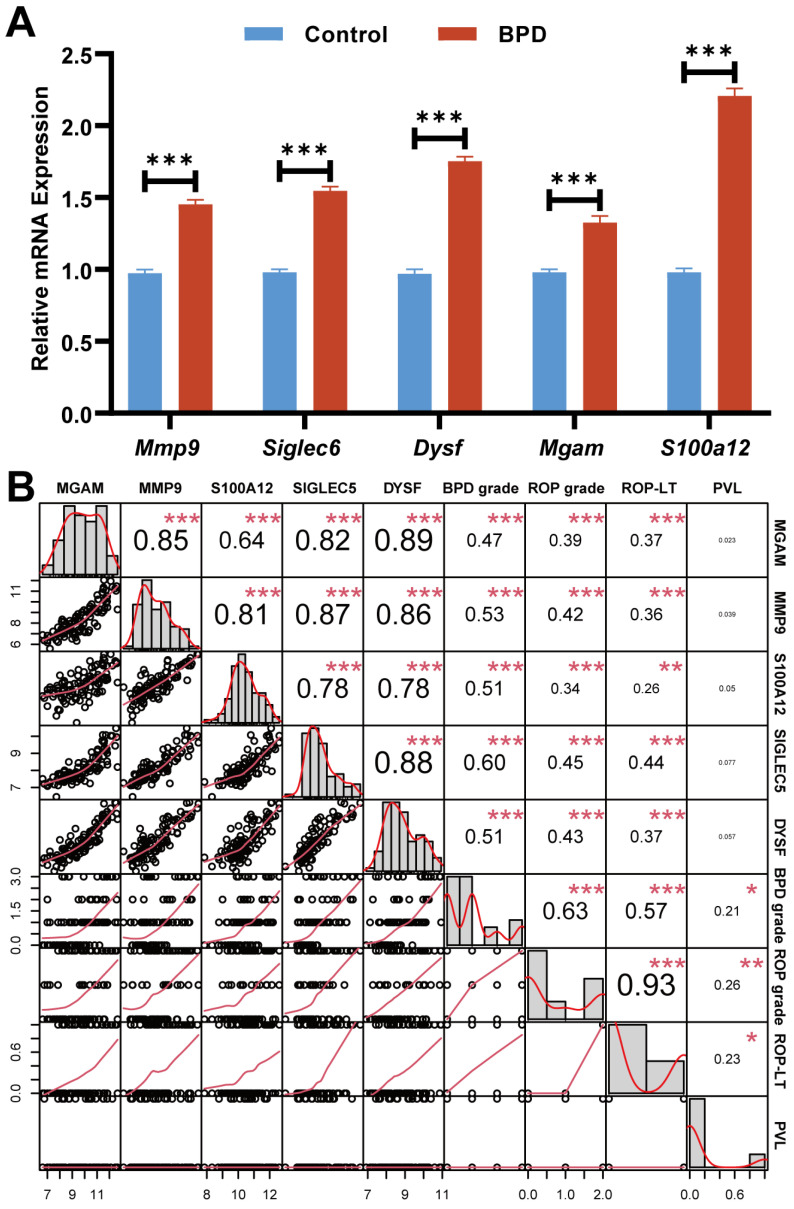
Relationship between the 5 biomarkers and patients’ clinical characteristics. (**A**) Expression levels of 5 biomarkers in peripheral blood of BPD neonates and controls. (**B**) The diagonal graph shows the distribution of the data in logarithmic ratios. The lower graph shows a scatter plot of the data, while the upper graph shows the correlation coefficient (r). Significant correlations are shown in numerical magnitude. Data were analyzed using Spearman’s correlation analysis. ROP: Retinopathy of prematurity, ROP-LT: Retinopathy of prematurity-laser therapy, PVL: Periventricular leukomalacia. Notes: * *p* < 0.05, ** *p* < 0.01, *** *p* < 0.001.

Finally, we collected clinical peripheral blood of BPD and control neonates to assess the mRNA levels of five biomarkers by using qRT-PCR. We found that all biomarkers were upregulated in the BPD group than controls (Figure 9A). The results were consistent with our bioinformatics analysis. (Figure 9A). To strengthen the clinical relevance of our findings, we further investigated the correlation between the five biomarkers and the clinical characteristics of BPD patients. Notably, all five biomarkers were significantly correlated with the grade of BPD, the grade of ROP, and the laser therapy of ROP. The expression of Siglec-5 was most highly correlated with the grade of BPD (r = 0.60, *p* < 0.001), and the expression of DYSF was most highly correlated with the grade of ROP and the laser therapy of ROP (r = 0.45/0.44, *p* < 0.001) (Figure 9B). In summary, these data suggest that the five biomarkers are associated with the development of BPD and its complications.

## 3. Discussion

In this comprehensive study, we employed integrated bioinformatics methods to investigate NET-related molecular signatures in BPD and identified five key biomarkers with potential diagnostic value. Differential expression analysis revealed 86 DEGs primarily involved in immune and inflammatory responses. This finding is consistent with the conclusions of Professor Kontomanolis’s research, emphasizing that in addition to causing the onset of preterm birth, inflammation is also a key factor in promoting multiple diseases in newborns, including bronchopulmonary dysplasia, retinopathy of prematurity, and necrotizing enterocolitis [10]. Enrichment analysis indicated a significant association of these genes with neutrophil extracellular trap formation and inflammatory pathways, underscoring the essential role of NETs in BPD pathogenesis. This provides favorable evidence for further exploration of molecular mechanisms.

A key finding of our study was the determination of 12 DENRGs through the intersection of DEGs, co-expression modules, and known NET-associated genes. The strong positive correlation between CXCR1 and CXCR2 in our analysis is particularly noteworthy, as these chemokine receptors are crucial for neutrophil recruitment and activation. The strong correlation between CXCR1 and CXCR2 (r > 0.8) reflects their biological relationship as IL-8 receptors with shared regulatory mechanisms and complementary functions in neutrophil chemotaxis and activation. In BPD context, this correlation is particularly significant as these receptors mediate neutrophil recruitment to the lungs, potentially driving inflammatory damage and abnormal lung development. In models of acute lung injury, overexpression of CXCR1/2 in induced pluripotent stem cell-derived endothelial cells significantly reduces lipopolysaccharide-induced acute lung injury, further highlighting the importance of CXCR1 and CXCR2 in inflammatory responses [11]. In addition, the correlation analysis showed primarily positive associations among the 12 DENRGs, with moderate correlations (0.4 < r < 0.7) observed between several gene pairs, including S100A12 with FPR2 and Siglec-5 with MMP9. These moderate correlations suggest coordinated but distinct functional relationships that may represent different aspects of neutrophil activity in BPD pathogenesis, such as neutrophil activation, degranulation, and extracellular trap formation pathways that contribute to lung injury.

The significant differences in immune cell proportions between BPD neonates and controls reflect the complex immune dysregulation underlying BPD pathophysiology. As Hansbro et al. reported, BPD involves dysregulation of key immune cells, including neutrophils, macrophages, and T cells, which contribute to inflammation, lung injury, and impaired lung development through aberrant immune responses [12]. Our analysis confirmed these findings, showing notably increased proportions of neutrophils, dendritic cells, and macrophages, along with reduced B cells, CD4+, and CD8+ T cells in BPD patients. Neutrophils, showing the most significant elevation, contribute to lung injury through release of proteases, reactive oxygen species, and extracellular traps that damage developing alveoli and airways. The reduction in T cells suggests impaired adaptive immunity, potentially disrupting the balance between pro- and anti-inflammatory responses critical for normal lung development. Increased MDSCs may contribute to immunosuppression and aberrant tissue remodeling, while alterations in monocytes and macrophages likely affect surfactant metabolism and resolution of inflammation. B cell reductions could impair antibody production, increasing susceptibility to infections that exacerbate lung injury. This immune landscape is consistent with recent studies emphasizing the importance of innate immunity in BPD, and the correlation between DENRGs and specific immune cell populations suggests that NET-related genes may influence BPD pathogenesis through modulation of the immune microenvironment.

Through the application of machine learning methods, we screened five robust biomarkers (MMP9, Siglec-5, DYSF, MGAM, and S100A12) for BPD diagnosis. Among these biomarkers, matrix metallopeptidase 9 (MMP9) has been extensively studied in lung pathology. Wu et al. demonstrated that increased MMP9 expression in tracheal aspirates of preterm infants was strongly associated with BPD development, and its elevation preceded clinical diagnosis by several days [13]. Additionally, Bhandari et al. revealed that MMP9 promotes aberrant lung remodeling in BPD through degradation of extracellular matrix components and disruption of alveolar development [14]. S100A12, a member of the S100 protein family, plays a crucial role in inflammatory responses. Liu et al. demonstrated that S100A12 promotes inflammation, oxidative stress, and cell apoptosis in sepsis-induced ARDS by activating the NLRP3 inflammasome pathway, suggesting it as a potential biomarker for diagnosing pulmonary injury in sepsis [15]. However, Liu et al. investigated the role of the inflammatory marker S100A12 in very premature infants, finding that its levels were significantly lower in those with respiratory distress syndrome compared to those without and do not differ between infants with or without BPD [16]. In this study, the expression level of S100A12 in two BPD validation datasets (5 days and 14 days after birth) was higher than that in the control group, indicating that S100A12 is a very important pathogenic factor in the entire BPD pathogenesis. In addition, the qRT-PCR validation results on clinical peripheral blood of neonates showed that S100A12 is also an effective diagnostic biomarker for BPD. Sialic acid binding Ig-like lectin 5 (Siglec-5), while not previously associated with BPD, has important immunoregulatory functions. Varki et al. revealed that the polymorphic paired receptors Siglec-5 and Siglec-14 modulate neutrophil and amnion responses to group B streptococcus, with Siglec-14 counteracting immune suppression by GBS, and highlight the impact of a Siglec-14-null polymorphism on susceptibility to infection and potential risk for prematurity [17]. Furthermore, Holownia et al. highlighted that inhaled corticosteroids increase the expression of Siglec-5/14 in sputum cells of COPD patients, with a particular focus on the immunosuppressive effects in patients who showed enhanced Siglec-5 expression, potentially contributing to immune modulation and suppression in these individuals [18]. Dysferlin (DYSF) is a membrane-associated protein involved in the repair of damaged cell membranes, particularly in muscle and epithelial tissues, and is characterized by its role in membrane fusion and calcium-dependent processes essential for maintaining cellular integrity and function [19]. Hackett et al. found that primary human airway epithelial cells express dysferlin, and through confocal microscopy, they further localized it within the Golgi, cell cytoplasm, and plasma membrane of 16HBE cells. However, it was not expressed in fibroblasts isolated from the bronchi and parenchyma [20]. Although this is a preliminary exploration, it lays the foundation for future research on the role of DYSF in BPD. Maltase-glucoamylase (MGAM) has not been previously linked to BPD or lung development. However, studies have shown its involvement in metabolic processes. Naim et al. elucidated the pivotal role of MGAM in carbohydrate metabolism and energy homeostasis [21]. Meanwhile, BPD is associated with metabolic dysregulation, as altered nutrient metabolism also affects lung development and impaired function in infants. The high predictive accuracy of our nomogram model (AUC = 0.807 in validation set 1) suggests these biomarkers could be valuable for early BPD diagnosis, particularly in the first few days after birth when intervention might be most effective. The selected biomarkers demonstrated solid diagnostic capacity for predicting bronchopulmonary dysplasia in premature infants, with an overall AUC of 0.75, indicating our panel correctly classifies BPD status with 75% accuracy, significantly better than random chance. This performance level represents clinically valuable predictive ability, particularly impressive given BPD’s complex, multifactorial nature and the heterogeneity of premature infant populations [22].

The identification of two distinct molecular clusters with different immune microenvironment features represents another important finding. Cluster C2 was identified as the “typical disease subtype” based on several converging lines of evidence. The molecular signature of C2 aligned with established BPD pathophysiology, featuring significantly upregulated expression of key neutrophil-related genes (Siglec-5, DYSF, MGAM, MMP9, and S100A12), which are known mediators of inflammatory lung injury. Additionally, the enriched pathways in C2 provide key insights into BPD mechanisms. The asthma pathway enrichment explains shared inflammatory processes and the increased risk of reactive airway disease in BPD survivors. Primary immunodeficiency pathway upregulation reflects compromised immune function in premature infants, contributing to recurrent infections that worsen lung injury. The intestinal immune network for IgA production pathway highlights the significant lung–gut axis in BPD, suggesting disrupted mucosal immunity and altered microbiome interactions may influence disease development, supporting potential therapeutic approaches targeting both pulmonary and intestinal inflammation. Furthermore, our drug–gene interaction analysis revealed potential therapeutic targets. In particular, MMP9 can be inhibited by MARIMASTAT and ANDECALIXIMAB; MGAM can be inhibited by ACARBOSE, MIGLITOL, and VOGLIBOSE. Based on our drug–gene interaction analysis, the inhibitory activity of the identified compounds against MMP9 and MGAM can be understood through predicted intermolecular interactions typical of small-molecule inhibitors. These likely involve hydrogen bonding with catalytic residues, hydrophobic interactions with binding pocket regions, and potential ionic bonds that collectively disrupt normal protein function. The effectiveness of these inhibitors depends on their structural complementarity with target proteins, where compounds with optimal spatial arrangement of functional groups can form stronger and more numerous bonds, resulting in higher binding affinity and greater inhibitory potency. While we did not perform molecular docking in this study, these established principles of protein–ligand interactions provide a foundation for understanding how the identified compounds may interfere with the biological activities of MMP9 and MGAM that contribute to bronchopulmonary dysplasia pathogenesis. The validation of our findings using qRT-PCR in clinical samples further strengthens the reliability of our bioinformatics analysis. In addition, the biomarkers were also closely related to the clinical features of BPD and its complications. The identification of Siglec-5 and DYSF as biomarkers highly correlated with both BPD and ROP severity reveals shared pathogenic mechanisms between these prematurity complications and offers valuable clinical applications. Siglec-5, an inhibitory receptor on neutrophils, likely represents a compensatory response to excessive inflammation, while DYSF’s role in membrane repair reflects ongoing tissue damage and repair processes, both marking injury in oxygen-vulnerable tissues. These correlations have significant clinical utility as predictive tools for identifying high-risk infants, guides for personalized treatment approaches, monitoring tools for assessing disease progression and treatment efficacy, potential dual-targeting therapeutic opportunities for both conditions, and research tools advancing our understanding of the molecular links between pulmonary and ophthalmologic complications of prematurity. This integrated biomarker approach represents a significant advancement toward more precise diagnosis, monitoring, and treatment of prematurity-related complications through targeted molecular pathways.

Several limitations of our study should be noted. First, while our findings are statistically robust, they are primarily based on transcriptomic data and require further functional validation. Second, the temporal dynamics of NET-related gene expression during BPD progression warrant further investigation. Third, the potential therapeutic implications of targeting the identified biomarkers need to be evaluated in appropriate experimental models.

## 4. Materials and Methods

### 4.1. Peripheral Blood Collection from Clinical BPD Patients

Bronchopulmonary dysplasia is diagnosed according to the diagnostic criteria published by the National Institute of Child Health and Human Development (NICHD) in 2001, when an infant requires oxygen support (inspired oxygen concentration (FiO_2_) > 21%) for more than 28 days after premature birth [23]. Peripheral blood samples discarded after routine laboratory testing were obtained from 10 BPD patients and 10 non-inflammatory patients between April and October 2024, with approval from the Ethics Committee of Sir Run Run Shaw Hospital, Zhejiang University School of Medicine (Approval No. 2024-2117-01).

### 4.2. Gathering BPD Data from Gene Expression Omnibus (GEO) Database

The GSE32472 dataset was acquired from GEO database (https://www.ncbi.nlm.nih.gov/geo/, accessed on 1 January 2025). Between 2008–2010, researchers in Poland analyzed the GSE32472 dataset comprising very low birthweight preterm infants (≤1500 g) requiring respiratory assistance at enrollment. Their main goal was to develop BPD endotypes using gene expression profiles obtained from whole blood microarray analysis. According to the blood collection schedule, the dataset had three time points: postnatal days 5, 14, and 28. This study analyzed 294 blood samples from 111 children after removing those with missing data: 62 BPD and 35 control samples from postnatal days 5, 58 BPD and 39 control samples from postnatal days 14, and 62 cases of BPD and 38 control samples from postnatal days 28. Based on the diagnostic criteria for BPD, we selected the 28-day postpartum dataset as the training group and the 5-day and 14-day postpartum datasets as the validation group. In addition, the dataset also contains several clinical parameters, such as the grade of BPD, the grade of ROP, whether laser therapy for ROP was received, and the presence of periventricular leukomalacia. Furthermore, 69 NET-related genes (NRGs) were sourced from a prior study [24].

### 4.3. Gene Expression and Functional Enrichment Analyses in BPD

Gene expression differences between BPD and control preterm infants were investigated using the “limma” package. Differential expression in BPD was attributed to genes as |log2FC| > 0.8 and a *p*-value < 0.05 [25]. Subsequently, the differentially expressed neutrophil extracellular traps (NET)-related genes (DENRGs) were acquired for subsequent examination. Significant DENRGs were mapped to chromosomal positions using the “RCircos” package and Spearman’s correlation coefficients. Heatmaps and volcano plots of DEGs were constructed with the R packages “pheatmap” and “ggplot2”. The R package “clusterProfiler” was employed to undertake the analysis of the biological functions of DEGs via Gene Ontology (GO) and Kyoto Encyclopedia of Genes and Genomes (KEGG) pathway enrichment [26].

### 4.4. Weighted Gene Co-Expression Network Analysis (WGCNA) in BPD

The WGCNA is a systems biology approach that identifies clusters of highly correlated genes (modules) by transforming gene expression data into a network where connections between genes are weighted based on expression correlation strength. The method uses hierarchical clustering to detect gene modules, summarizes each module with a representative eigengene, and correlates these modules with clinical traits to identify candidate biomarkers and gene sets associated with specific disease phenotypes. Co-expression networks of the GSE32472 dataset were constructed via the WGCNA approach in accordance with the scale-free topology. The soft threshold power and adjacencies were calculated by employing the pickSoftThreshold within “WGCNA” package [27]. The adjacency matrix was employed to generate a topological overlap matrix, and a computation of dissimilarity was conducted to establish the results of hierarchical clustering. To identify co-expressed gene modules, a dynamic tree-cutting technique was applied, requiring a minimum of 10 genes per module. The primary module linked to BPD patients was ascertained by evaluating the values of gene significance and module membership.

### 4.5. Immune Cells Infiltrating in BPD

The differential abundances of 28 immune-infiltrating cells were computed using the ssGSEA algorithm by “gsva” package. Immune cell abundances were visualized through a heatmap and violin plot, generated with R packages of “corrplot” and “ggplot2”. The correlations between DENRGs and immune cells were evaluated using Spearman’s correlation coefficients with the R package “ggpubr”.

### 4.6. Identifying Key Biomarkers in BPD

Biomarkers in the training datasets were selected using the LASSO binary logistic regression model implemented in the “glmnet” package [28]. A 10-fold cross-validation process was applied to determine the best penalty parameter for each signature. Biomarker identification was conducted using the “e1071”, “kernlab”, and “caret” packages through a support vector machine recursive feature elimination (SVM-RFE) method with a nonlinear SVM [29]. A random forest model was employed to create 500 trees per data point, identifying biomarkers for BPD with DecreaseGini score exceeding 2 [30].

### 4.7. Building and Validating the Column Line Plot Model

To evaluate the risk of BPD clustering, a column line plot model was established with “rms” package. A score was assigned to each predictor, and the total score was the cumulative sum of these scores. Calibration curves and decision curve analysis (DCA) were applied to assess the model’s predictive accuracy (39617734). Box plots illustrating the expression of key biomarkers in BPD and control neonate testing sets were generated using the “ggpubr” and “ggplot2” packages. The “pROC” package facilitated the computation of receiver operating characteristic (ROC) curves, with the area under the curve (AUC) assessing the predictive values of biomarkers [31].

### 4.8. Unsupervised Clustering and Gene Set Variation Analysis (GSVA) Analysis of BPD

Expression profiles of 5 biomarkers were clustered using the “ConsensusClusterPlus” package with 1000 iterations in an unsupervised manner [32]. The CDF curve served as a key metric for evaluating the stability of our consensus clustering analysis and determining the optimal number of BPD subtypes. The k-means algorithm was used to cluster 62 BPD samples into distinct groups. The optimal cluster count was identified by evaluating the cumulative distribution function (CDF) curve and consensus matrix, ensuring a cluster-consensus score exceeding 0.9, with the maximum subtypes ranging from 2 to 9 [33]. Principal component analysis (PCA) was carried out to emphasize the distinctions between subtypes [34]. Differential pathway analyses were executed by employing the “GSVA” and “GSEABase” packages for the purpose of recognizing variations in enriched gene sets among distinct clusters.

### 4.9. Drug-Gene Interaction Insights

This study employed the DGIdb method (https://dgidb.org, accessed on 10 January 2025) to forecast possible drug interactions for screened biomarkers, establishing a foundation for targeted therapeutic interventions. The drug–gene interactions network was displayed using Cytoscape (version 3.9.1).

### 4.10. qRT-PCR in Peripheral Blood of BPD

PBMCs from clinical patients were isolated by whole blood. Total mRNA was extracted from tissues using TRIzol-chloroform (Invitrogen, Carlsbad, CA, USA). The cDNA was synthesized via reverse transcription utilizing PrimeScript RT reagent Kit (RR037A, TaKaRa, Shiga, Japan). PCR analysis was conducted using 7900HT Real-Time PCR system (Applied Biosystems, Waltham, MA, USA). Ultimately, the fold change was calculated using the formula 2^(−ΔΔCt)^. The qRT-PCR primers are summarized in Table 1.

### 4.11. Statistical Analysis

Statistical computations were executed with the R software (version 4.0.2). The Wilcoxon rank-sum test was utilized to compare group discrepancies. Spearman’s correlation analysis determined both the correlation between DEFRG and immune cells and the relationship between the biomarkers and the clinical characteristics of patients. A two-tailed *p*-value less than 0.05 was selected to determine statistical significance.

## 5. Conclusions

In conclusion, this research identified key NRGs involved in bronchopulmonary dysplasia, revealing their role in immune dysregulation. The clinical predictive ability of five biomarkers (MMP9, Siglec-5, DYSF, MGAM, and S100A12) for the development of BPD was validated, and drug–gene interaction analysis highlighted MGAM and MMP9 as potential therapeutic targets. These findings improve our comprehension of BPD pathogenesis and present promising opportunities for precision diagnostics and targeted therapy.

## Figures and Tables

**Table 1:** Primers applied to quantitative real-time PCR.

Primers	Sequence (5′->3′)	
MMP9	Forward	5′-TGTACCGCTATGGTTACACTCG-3′
	Reverse	5′-GGCAGGGACAGTTGCTTCT-3′
Siglec-5	Forward	5′-TTCAGGAACGGCATAGCCCTA-3′
	Reverse	5′-TACTCGACGAAGCTCCAAGAT-3′
DYSF	Forward	5′-AAGAACAGCGTGAACCCTGTA-3′
	Reverse	5′-CCTCTCGGAGTGGGACCTT-3′
MGAM	Forward	5′-GCTCAGTGTTCTTCTGCTTGT-3′
	Reverse	5′-CGTTGTCCTAGCATGTGTGGTA-3′
S100A12	Forward	5′-AGCATCTGGAGGGAATTGTCA-3′
	Reverse	5′-GCAATGGCTACCAGGGATATGAA-3′
GAPDH	Forward	5′-GCCTCAAAATCCTCTCGTTGTG-3′
	Reverse	5′-GGAAGATGGTGATGGGATTTC-3′

## Data Availability

The datasets supporting the conclusions of this article are available in the GEO database (https://www.ncbi.nlm.nih.gov/geo/query/acc.cgi?acc=GSE32472, accessed on 1 January 2025).
